# Environmental enrichment does not influence hypersynchronous network activity in the Tg2576 mouse model of Alzheimer’s disease

**DOI:** 10.3389/fnagi.2015.00178

**Published:** 2015-09-23

**Authors:** Charlotte Bezzina, Laure Verret, Hélène Halley, Lionel Dahan, Claire Rampon

**Affiliations:** ^1^UMR5169 CNRS, Centre de Recherches sur la Cognition Animale, Université de Toulouse, Université Paul SabatierToulouse, France; ^2^CNRS, Centre de Recherches sur la Cognition AnimaleToulouse, France

**Keywords:** amyloid precursor protein, EEG, epileptiform activity, pentylenetetrazole, seizure susceptibility, Alzheimer’s disease, environmental enrichment

## Abstract

The cognitive reserve hypothesis claims that the brain can overcome pathology by reinforcing preexistent processes or by developing alternative cognitive strategies. Epidemiological studies have revealed that this reserve can be built throughout life experiences as education or leisure activities. We previously showed that an early transient environmental enrichment (EE) durably improves memory performances in the Tg2576 mouse model of Alzheimer’s disease (AD). Recently, we evidenced a hypersynchronous brain network activity in young adult Tg2576 mice. As aberrant oscillatory activity can contribute to memory deficits, we wondered whether the long-lasting memory improvements observed after EE were associated with a reduction of neuronal network hypersynchrony. Thus, we exposed non-transgenic (NTg) and Tg2576 mice to standard or enriched housing conditions for 10 weeks, starting at 3 months of age. Two weeks after EE period, Tg2576 mice presented similar seizure susceptibility to a GABA receptor antagonist. Immediately after and 2 weeks after this enrichment period, standard and enriched-housed Tg2576 mice did not differ with regards to the frequency of interictal spikes on their electroencephalographic (EEG) recordings. Thus, the long-lasting effect of this EE protocol on memory capacities in Tg2576 mice is not mediated by a reduction of their cerebral aberrant neuronal activity at early ages.

## Introduction

Alzheimer’s disease (AD) is the first cause of dementia worldwide. AD patients present a progressive decline of memory performances which amplitude and speed are highly variable between individuals. Notably, environmental factors can modulate the risk of dementia in AD and a high educational level or the practice of various leisure activities has been associated with a decreased risk of AD-related dementia (Stern et al., 1994; Scarmeas et al., 2001). The concept of cognitive reserve hypothesizes that brain adapts to overcome pathology by reinforcing the efficiency of usual cognitive strategies and the development of alternative ones. Given the poor efficiency of current therapeutic strategies against AD, it has become a major issue to better understand memory decline during the course of the pathology and to decipher the neurobiological basis of cognitive reserve.

Environmental enrichment (EE) is an experimental paradigm providing social, sensorial and cognitive stimulations for animals that mimics the beneficial human lifestyle. It thus constitutes a unique tool to investigate the biological processes involved in the establishment of the cognitive reserve. EE consists in housing large groups of animals in a wide cage filled with objects of different size, shape, color and material that are regularly renewed (Sztainberg and Chen, 2010). Exposure to EE has extensively been reported to improve cognitive performances in wild-type mice (Rampon et al., 2000; Tang et al., 2001; Frick and Fernandez, 2003; Jankowsky et al., 2005; Berardi et al., 2007; Costa et al., 2007; Cracchiolo et al., 2007; Dong et al., 2007; Gerenu et al., 2013). In mouse models of AD, memory performances were found to be improved immediately after long-lasting exposure to EE (more than 4 months; Jankowsky et al., 2005; Berardi et al., 2007; Costa et al., 2007; Cracchiolo et al., 2007; Dong et al., 2007). More recently, we reported that an early and transient exposure to EE leads to a long-lasting effect on memory function in the Tg2576 mouse model of AD (Verret et al., 2013). Tg2576 mice, which express the Swedish double mutant form of the human Amyloid Precursor Protein (APP), progressively develop AD-related behavioral and neuroanatomical troubles (Hsiao et al., 1996). In this mouse line, memory deficits appear around 3 months of age (Jacobsen et al., 2006; D’Amelio et al., 2011; Duffy et al., 2015) and progress slowly while amyloid plaques form later, around 10 months of age (Kawarabayashi et al., 2001). We showed that exposure to EE that occurs between 3 and 5.5 months of age, improved memory performances of 13 month-old Tg2576 mice, while Tg2576 mice housed in standard cages displayed robust memory deficits (Verret et al., 2013). These results corroborate data in human revealing that education, an early-life cognitive stimulation, delays the onset of AD-type dementia in the elderly (Stern et al., 1994). Our EE protocol is thus a suitable paradigm to study the neural mechanisms underlying the formation of cognitive reserve during young adulthood in mice. Interestingly, if Tg2576 mice underwent EE after 5 months of age, we observed that the beneficial effects on memory function were less robust across memory tasks (Verret et al., 2013). We thus hypothesized that EE taking place at 3 months of age could disrupt one or several early pathogenic events of amyloidopathy, before their deleterious effects on brain function and memory processing become permanent, thereby durably limiting memory alterations. This hypothesis would explain why an EE exposure later during the disease time-course was not or less efficient to rescue memory performances in Tg2576 mice (Verret et al., 2013).

Amongst early events linked to AD pathogenesis that can contribute to memory decline, we focused here on neuronal network hypersynchrony (Bakker et al., 2012; Vossel et al., 2013). Indeed, seizures are more frequent in AD patients than in age-matched individuals and seizures can precede the onset of memory deficits (Amatniek et al., 2006; Sanchez et al., 2012). Different lines of evidence also indicate the occurrence of hypersynchronous network activity such as seizures amongst mouse models of AD (Palop et al., 2007; Minkeviciene et al., 2009; Born et al., 2014; Ittner et al., 2014). Interestingly, preventing neuronal hyperactivity with the anti-epileptic drug levetiracetam was associated with an improvement of memory performances in subjects with Mild Cognitive Impairments and in the hAPPJ20 mouse model of AD (Bakker et al., 2012; Sanchez et al., 2012). Altogether these data strongly suggest that aberrant neuronal activity can contribute to memory dysfunction in AD. Recently, we evidenced that network hypersynchrony occurs at very early age in Tg2576 mice (Bezzina et al., 2015). Tg2576 mice present a higher susceptibility to pharmacologically-induced seizures than their non-transgenic (NTg) littermates. They also display an epileptiform activity in the form of frequent interictal spikes on their electroencephalograms (EEG), high-amplitude events that typically occur between seizures in epileptic patients and rodents, even if no seizure was recorded over the recording time. This network hypersynchrony was observed in Tg2576 mice as soon as 1.5 months of age that is to say before the onset of memory decline in this mouse line. Therefore, hypersynchronous network activity could be part of the earliest pathogenic events leading to memory decline in Tg2576 mice, which may be prevented by EE.

Yet, EE can suppress seizures or reduce seizure susceptibility in rat models of epilepsy (Young et al., 1999; Auvergne et al., 2002; Korbey et al., 2008; Fares et al., 2013). For instance, housing rats in enriched environment before pharmacological or electrical induction of seizures reduced the severity of subsequent seizures (Young et al., 1999; Auvergne et al., 2002). In a genetic mouse model of epilepsy, enriched housing starting at birth totally suppressed seizures (Manno et al., 2011).

In the present work, we examined whether an EE protocol taking place between 3 and 5.5 months of age, that has proven long-lasting effect on memory function in Tg2576 mice, could reduce network hypersynchrony in these mice. To address this question, we performed *in vivo* EEG recordings to detect interictal spikes and assessed seizure susceptibility to a GABA receptor antagonist in Tg2576 females and their NTg littermates housed under standard or enriched conditions.

## Materials and Methods

### Ethics Statement

As previously described (Bezzina et al., 2015), all experiments were performed in strict accordance with the policies of the European Union (86/609/EEC), the French National Committee of Ethics (87/848), and the local committee’s recommendations (C 31-555-11, Direction départementale de la protection des populations) for the care and use of laboratory animals. Animal facility of the Centre de Recherches sur la Cognition Animale (CRCA) is fully accredited by the French Direction of Veterinary Services (C 31-555-11, February 9, 2011) and animal surgery and experimentation conducted in this study were authorized by the French Direction of Veterinary Services (#31-11555521, 2002). All efforts were made to improve the mice welfare and minimize their suffering.

### Mouse Line

As described in Bezzina et al. (2015), experiments were performed on female mice from our in-house colony of the transgenic line Tg2576 (Hsiao et al., 1995, 1996). Tg2576 mice overexpress a double mutant form of human APP695 (Lys670-Asn, Met671-Leu [K670N, M671L]), driven by a hamster prion protein promoter. Tg2576 males were bred with C57B6/SJL F1 females (Charles River, L’Arbresle, France) and the offspring was genotyped for the hAPP transgene using DNA obtained from post-weaning tail biopsies. Polymerase chain reaction products were analyzed to confirm the presence of hAPP DNA sequence in offspring. Mice were maintained on a 12 h light/12 h dark cycle with free access to food and water.

### Environmental Enrichment

**Figure 1:**
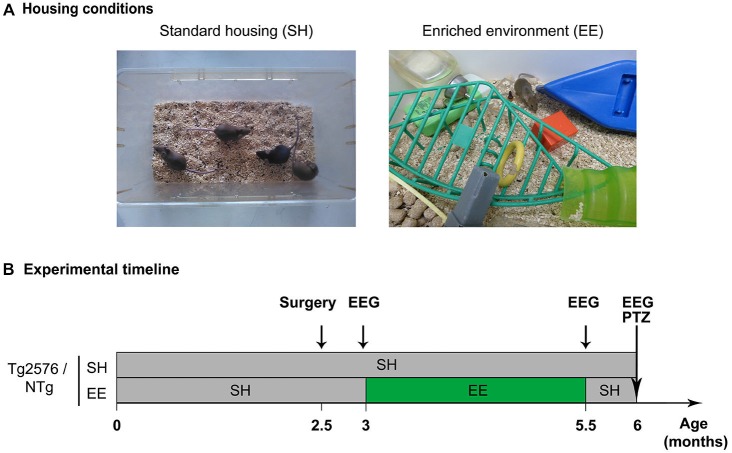
**Housing conditions and experimental plan. (A)** Photographs illustrating a standard laboratory cage (left; standard housing, SH) and an enriched environment (EE; right). **(B)** Experimental timeline depicting the mouse groups, housing conditions, enrichment period and experimental schedule. Abbreviations: SH, Standard Housing; EE, Environmental Enrichment; EEG, Electroencephalography; PTZ, Pentylenetetrazole; NTg, non-transgenic.

At 3 months of age, Tg2576 and NTg mice were arbitrary divided in two groups. One group (SH) was housed in standard laboratory cages, by lots of 2–5 mice, until the age of 6 months (Figure 1A, left). The other group was housed in an enriched environment (EE; Figure 1A, right), as previously described (Verret et al., 2013) until mice reached the age of 5.5 months after which they were returned to their standard cages until the age of 6 months (Figure 1B). Whatever the housing conditions, mice were given ad libitum access to food and water. As previously described (Verret et al., 2013), EE was composed of a large box (150 × 80 × 80 cm) containing various objects of different size, shape, color and material (wood, plastic, glass, metal), excluding running wheels. The configuration of the objects was modified and new objects were introduced every other day in order to stimulate mice exploratory behavior. Mice were exposed to the enriched environment by groups of 7–12 individuals (Figure 1A, right).

### Implantation of EEG Electrodes

Tg2576 mice and NTg littermates were implanted with electrodes for EEG recordings at the age of 2.5 months (Figure 1B; *NTg EE*: *n* = 10, *Tg2576 EE*: *n* = 11, *NTg SH*: *n* = 13, *Tg2576 SH*: *n* = 11) as previously described in details (Bezzina et al., 2015).

### EEG Recordings

Mice performed EEG recordings at three time-points: 3, 5.5 and 6 months of age, i.e., respectively 1–5 days before, immediately after, or 2 weeks after the EE period (Figure 1B). Mice were habituated to the recording system 1–5 days before the first recording session (at 3 months of age). Each recording session lasted 24 h. Protocols and device used for habituation and EEG recordings were the same as previously detailed (Bezzina et al., 2015). EEG and EMG signals were amplified and band-pass filtered (for EEG: 0.3–1 KHz; for EMG: 3 Hz-20 kHz) using a AM system 3500 amplifier (A-M system, Sequim, WA, USA) and sampled at 200 Hz (Power 1401 mk-II, CED, Cambridge, UK). EEG recordings were analyzed using Spike 2 V7 software.

### Detection of Epileptiform Activity on EEG Traces

Interical spikes were visually detected on EEG traces based on metrical, morphological and temporal criteria (Bezzina et al., 2015). The number of interictal spikes per minute was quantified over the 24 h of recordings. One mouse died between 3 and 5.5 months and was removed from the analysis for all ages (*Tg2576 SH*: *n* = 1) and we excluded another animal because of movements artifacts on EEG traces (*NTg SH*: *n* = 1). Statistical analysis of the frequency of interictal spikes (number of spikes/minute) was performed using a two-way ANOVA for repeated measures, followed by a Bonferroni *post hoc* test.

### PTZ Injection

Seizure susceptibility was evaluated by behavioral scoring of the severity of seizures induced by the injection of a GABAa receptor antagonist: pentylenetetrazole (PTZ, Sigma Aldrich, St Louis, MO, USA). Mice were administered with a single i.p. injection of PTZ at 40 mg/kg (*NTg EE*: *n* = 23, *Tg2576 EE*: *n* = 23, *NTg SH*: *n* = 29, *Tg2576 SH*: *n* = 24). Two mice were excluded from the analysis because they received a lower PTZ dose by accident (*Tg2576 EE*: *n* = 1, *NTg EE*: *n* = 1). After drug injection, each mouse was placed in a new cage and its behavior was videotaped for 20 min. Mice were sacrificed immediately after to limit suffering.

### Seizure Severity Scoring

As previously explained (Bezzina et al., 2015), the maximal seizure severity reached along the 20 min session was scored using an ordinal behavioral scale (Palop et al., 2007; Bezzina et al., 2015) as follows: “0 = normal exploratory behavior, 1 = immobility, 2 = generalized spasm, tremble, or twitch, 3 = tail extension, 4 = forelimb clonus, 5 = generalized clonic activity, 6 = bouncing or running seizures, 7 = full tonic extension, 8 = death”. Severity scores were validated by two independent observers blind to the experimental conditions and compared among all groups using Kruskal-Wallis test, followed by Dunn’s *post hoc* tests for side by side comparisons.

### Statistical Analysis

All statistical analysis was performed using the Prism 5 software (GraphPad Software Inc., La Jolla, CA, USA).

## Results

### Environmental Enrichment does not Affect Epileptiform Activity in Tg2576 Mice

**Figure 2:**
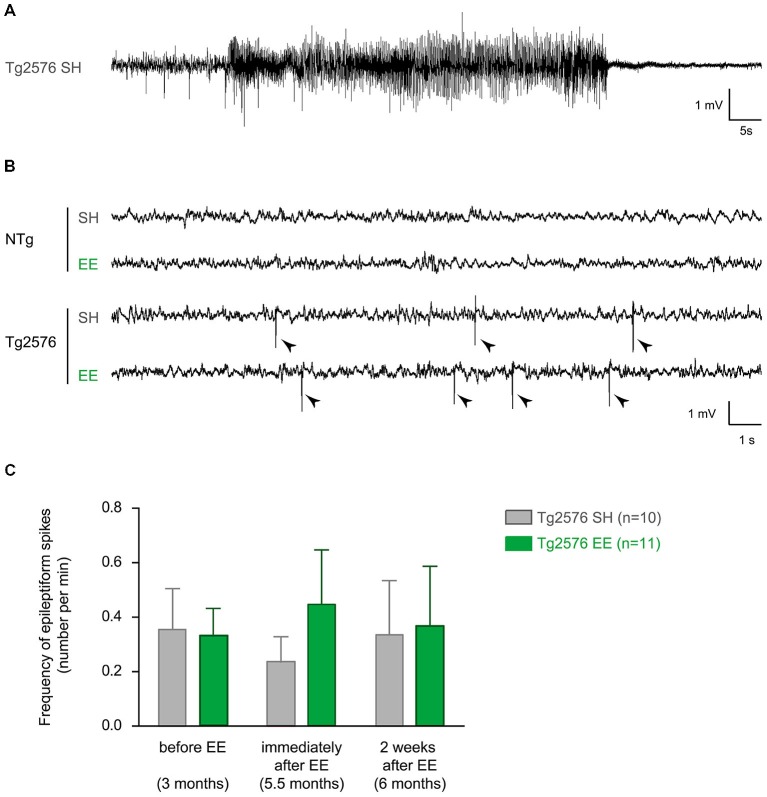
**Environmental enrichment does not influence interictal spike frequency in Tg2576 females. (A)** Representative electroencephalographic (EEG) trace of a seizure recorded in a Tg2576 mouse (3 months of age) housed under standard conditions. It is characterized by a high amplitude and high frequency oscillation lasting several seconds, followed by regular low-amplitude oscillation. **(B)** Representative EEG traces of 6 month-old non-transgenic (NTg; top) and transgenic (bottom) mice housed under standard (SH) and enriched conditions (EE). Note that only Tg2576 mice display frequent interictal spikes (sharp, high-amplitude events indicated by arrow heads). **(C)** Quantitative analysis of the frequency of interictal spikes (mean ± SEM) in Tg2576 mice housed in standard laboratory cages (SH, *n* = 10) or in enriched environment (EE, *n* = 11), before (at 3 months), immediately after (5.5 months) and 2 weeks after the EE period (6 months). NTg mice are not represented since they do not display any spike whatever the housing conditions or age. A two-way ANOVA for repeated measures shows no effect of recording time (*p* = 0.99), no effect of housing conditions (*p* = 0.73) and no interaction (*p* = 0.43).

To measure the acute and durable effect of EE on the epileptiform activity in Tg2576 mice, we performed three EEG recording sessions of 24 h each on Tg2576 mice and their NTg littermates. For each mouse, these recordings took place: (i) 1–5 days before EE (at the age of 3 months); (ii) immediately after EE (at the age of 5.5 months); and (iii) 2 weeks after EE (at the age of 6 months; Figure 1B). As previously described (Bezzina et al., 2015), we did not observe any spike in NTg mice whatever their age or housing conditions. In Tg2576 mice, we recorded three seizures over the 22 animals. One seizure was recorded at 3 months of age in a Tg2576 mouse (Figure 2A) that died 2 weeks after. This Tg2576 mouse was housed in standard conditions. The two other seizures were both recorded in one Tg2576 mouse housed under standard conditions. One seizure occurred during the recording session at 5.5 months of age, the other one during the recording session that took place 2 weeks after. All Tg2576 mice exhibited interictal spikes (Figure 2B) which frequency did not vary across recording sessions nor housing conditions (Figure 2C; two-way ANOVA for repeated measures; age effect: *p* = 0.99; housing effect: *p* = 0.73; interaction: *p* = 0.43). Environmental enrichment did not modify EEG oscillations either. Indeed, for both genotypes, power spectra calculated from 1–100 Hz for each vigilance state showed no difference between housing conditions at any frequency (two-way ANOVA, *p* > 0.86 for the effect of housing conditions and for the interaction between frequency and housing conditions). These results reveal that EE at early age does not affect epileptiform activity in Tg2576 mice.

### Environmental Enrichment has no Lasting Effect on the Susceptibility to Pharmacologically-Induced Seizures in Tg2576 Mice

**Figure 3:**
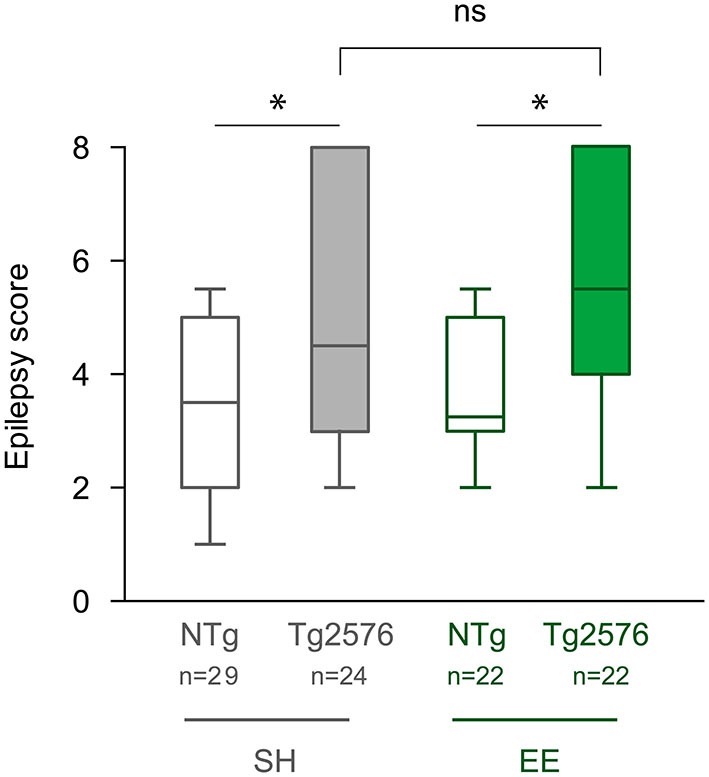
**Environmental enrichment does not modify the susceptibility to PTZ-induced seizures in Tg2576 females.** Seizure severity score of 6 month-old Tg2576 and non-transgenic (NTg) females, housed under standard (SH) or enriched conditions (EE). Whiskers boxes represent the interquartile distribution. Kruskal-Wallis test reveals a significant global effect (*p* = 0.0012). ns: *p* = 0.05; *p < 0.05 for Dunn’s *post hoc* tests.

Our aim was to determine whether EE has an effect on seizure susceptibility in Tg2576 mice. After the completion of EE, one or 2 days after the last EEG recording, we assessed seizure susceptibility to the GABA_A_ receptor antagonist PTZ in NTg and Tg2576 mice housed in standard or enriched conditions. Tg2576 females exhibited more severe seizures than NTg females (Figure 3; Kruskal-Wallis: *p* = 0.0012, Dunn’s *post hoc* tests: *p* < 0.05 for NTg EE *vs* Tg2576 EE and for NTg SH *vs* Tg2576 SH). However, our data indicate that seizure severity did not differ between Tg2576 mice housed under standard and enriched conditions (Dunn’s *post hoc* test: *p* > 0.05 for Tg2576 SH *vs* Tg2576 EE). In summary, EE does not durably modify seizure susceptibility to PTZ in Tg2576 females.

## Discussion

Our work shows that EE performed between 3 and 5.5 months of age does not acutely affect EEG interictal spikes in the Tg2576 mouse model of AD. We also show that, 2 weeks after this EE protocol, Tg2576 mice exhibit a susceptibility to PTZ-induced seizures and a frequency of EEG interictal spikes that were similar to those observed in animals housed in standard conditions. Altogether, these observations show that the prevention of memory impairments by EE in Tg2576 mice is not due to a reduction of neuronal network hypersynchrony at early ages.

In a previous work, we described hypersynchronous network activity in Tg2576 mice at 6 months of age, in males (Bezzina et al., 2015). Spikes were virtually absent in both males and females NTg mice. In 6 month-old NTg mice housed in standard conditions, PTZ-induced seizures reported here in NTg females were significantly more severe than those previously reported in NTg males (1st quartile/ median/3rd quartile for females: 2/3.5/5 and males: 1/2/3.125, Mann and Whitney test *p* = 0.0138). In 6 month-old Tg2576 mice housed in standard conditions, PTZ-induced seizures and the frequency of interictal spikes were not significantly different between Tg2576 males and females (Mann and Whitney males *vs* females: PTZ severity score: *p* = 0.77, spike frequency: *p* = 0.7693). The female higher sensitivity to PTZ that we observed in NTg mice could be related to the fluctuations of GABA_A_ receptors composition along the oestrus cycle and the associated seizure susceptibility (Wu et al., 2013). This gender difference in PTZ sensitivity may be more obvious in NTg than in Tg2576 mice because of a ceiling effect in Tg2576 mice due to the ordinal nature of the severity scale. In summary, we observed a network hypersynchrony in Tg2576 female mice which was very similar to what we previously reported in males.

Our data reveal that there is no effect of EE on epileptiform activity in Tg2576 mice, immediately after the EE. Nevertheless, we could not exclude that EE acutely affects other parameters such as the frequency of seizures or their duration. However, if EE had a robust effect on network hypersynchrony, one would expect that it would have impacted the frequency of interictal spikes, which is not the case in our study.

Two weeks after EE, both the severity of PTZ-induced seizures and the frequency of interictal spikes were comparable between enriched and standard-housed Tg2576 mice. Thus, there is no noticeable lasting effect of EE on network hypersynchrony of Tg2576 mice. These results confirmed our previous data showing that neuropeptide Y ectopic expression in mossy fibers, a marker of chronic seizures, was present in standard-housed 13 month-old Tg2576 mice even if those mice had previously been housed in enriched environment at 3 months of age (Verret et al., 2013). Studies in rodent models of epilepsy have already assessed the effect of EE on their epileptic phenotype. Rats housed in enriched environment before undergoing an epileptogenic process induced by kainate injections or electrical stimulations developed fewer seizures than standard-housed rats (Young et al., 1999; Auvergne et al., 2002). Conversely, rats that received lithium/pilocarpine injections to trigger epileptogenesis before 1-month exposure to EE, showed similar spike rates than rats housed in standard conditions (Rutten et al., 2002). Moreover, EE carried at very early age (from P21 to P51) in Bassoon mutant mice, which developed seizures before P14, did not suppress seizures (Morelli et al., 2014). Thus, it seems that EE needs to occur before the induction of epileptogenesis in order to prevent it. In Tg2576 mice, hypersynchronous network activity is already observed at 1.5 months of age (Bezzina et al., 2015), i.e., prior the onset of EE in our study (3 months of age). Thus, the EE performed in our study might occur too late to prevent network hypersynchrony in Tg2576 mice.

Interestingly, the same EE protocol can produce different effects according to the origin of the epileptic phenotype. Schridde and Van Luijtelaar (2004) showed that the same protocol of EE increased or had no effect on the number of epileptic discharges in two strains of epileptic rats WAG/Rij and ACI, possibly because these two kinds of epilepsy relied on different physiopathological mechanisms. It is thus plausible that network hypersynchrony in Tg2576 mice is not suppressed by EE as in the aforementioned rodent models of epilepsy (Young et al., 1999; Auvergne et al., 2002; Manno et al., 2011) because the neurobiological basis of hypersynchronous network activity are different in AD and epilepsy models.

In the study by Verret et al. (2013), the same EE protocol performed between 3 and 5.5 months of age prevented memory impairments of 13 month-old Tg2576 mice whereas standard-housed Tg2576 mice showed clear memory deficits. In the spatial Morris Water Maze test, the Tg2576 mice previously enriched at 3 months of age swam preferentially in the target quadrant, similarly to NTg mice (Verret et al., 2013). Our work thus reveals that the beneficial long-lasting effect of an early EE on memory performances of Tg2576 mice is not mediated by a suppression of hypersynchronous network activity at early ages in this mouse line. Rutten et al. (2002) reported similar results in rats that were enriched after experimenting a *status epilepticus*. *Status epilepticus*-induced memory impairments were less pronounced in enriched rats than in standard-housed rats, while the frequency of interictal spikes was not significantly different between these two groups of rats (Rutten et al., 2002). We can thus hypothesize that EE has no influence on dementia pathogenesis but rather triggers general compensatory mechanisms to maintain memory performances in Tg2576 mice. Indeed, EE stimulates different pathways regulating brain plasticity that contribute to the effect of EE on memory processes in mice. These processes include increased neurogenesis, increased levels of neurotrophins or increased long-term potentiation (for review Nithianantharajah and Hannan, 2006). Gerenu et al. (2013) reported that a transient cognitive stimulation in early life (from 4–6 months of age) improved memory performances of Tg2576 mice at 15 months of age. They also reported an increased level of proteins involved in synaptic plasticity and memory formation such as the PSD95 postsynaptic scaffold protein, the NR1 NMDA receptor subunit and the immediate early gene Arc (Gerenu et al., 2013). Whether an increase of such proteins is responsible for the long-lasting memory improvement provided to Tg2576 mice by EE remains to be investigated.

## Conclusion

Environmental enrichment performed between 3 and 5.5 months of age in Tg2576 mice does not have any detectable effect on the mice seizure susceptibility to PTZ nor on interictal spike frequency. The long-lasting effect of EE on memory performances of Tg2576 mice is thus not mediated by a suppression of hypersynchronous network activity at early ages in this mouse line. Such early-life EE might exert its capacity to improve memory by interfering with other processes involved in memory dysfunction or by triggering compensatory mechanisms such as enhanced plasticity.

## Author Contributions

Conceived and designed the experiments: CB, LV, LD, CR. Acquired data: CB, HH, LD. Analyzed and interpreted the data: CB, LV, LD, CR. Wrote the manuscript: CB, LV, LD, CR.

## Funding

ANR-10-05-MALZ: CR, LD; Agence Régionale de Santé, France: CB, Subvention de recherche Fyssen 2013: LV.

## Conflict of Interest Statement

The authors declare that the research was conducted in the absence of any commercial or financial relationships that could be construed as a potential conflict of interest.

## Abbreviations

AD: Alzheimer’s disease
APP: Amyloid precursor protein
EE: environmental enrichment
NTg: non transgenic
PTZ: pentylenetetrazole
SH: standard housing(ed).
